# Supplementary material for: Clinical usefulness of brief screening tool for activating weight management discussions in primary cARE (AWARE): A nationwide mixed methods pilot study
Source: PLoS One. 2021 Oct 28;16(10):e0259220. doi: 10.1371/journal.pone.0259220 (PMC8553075; doi:10.1371/journal.pone.0259220)
Supplement: S1 Appendix — (DOCX) [file pone.0259220.s001.docx]

| **S1. Appendix.** **EOSS-2 Risk Screening Tool** |
| --- |

The Edmonton Obesity Staging System-2 Risk Tool

(EOSS-2 Risk Screening Tool)

**What are weight related health problems?**

Overweight and obesity is the greatest public health issue challenging Australia’s health care system affecting a quarter of all young people (aged 2-17 years) and around two-thirds of all adults. The most common weight related health problems include cancer, stroke, heart disease, kidney disease, diabetes, back pain, and osteoarthritis. There are approximately 7 million Australians with possible weight related complex and/or chronic conditions resulting in avoidable health service use including hospitalizations.^1^

**What are the barriers to accessing health care?**

While General Practitioners (GPs) can provide evidence-based recommendations on effective interventions, excess weight and related health problems remain under diagnosed and poorly treated. The vast majority of patients with overweight or obesity want their GP to bring up weight management during appointments, but they seldom do. People with excess weight are often stigmatised by others in the community, including by those providing health care. This can cause people with overweight or obesity to delay having a conversation about weight management with a health care professional.

**How can the EOSS-2 Risk tool enable access to health care**

The most important criterion GPs consider for initiating weight management conversations with a patient is if they have, or are at risk of developing new or additional, weight related health problems. This suggests that targeting weight related health status rather than obesity *per se* may overcome this barrier to initiating treatments in primary care. The EOSS is a clinically useful obesity staging system based on weight related health problems among individuals with overweight or obesity.^2,3^ A score of 2 or higher on the EOSS indicates the presence of clinically significant weight related health problems requiring clinical intervention.

The EOSS-2 is a brief diagnostic screening tool for predicting clinically significant weight related health problems in patients with excess weight based on confirmed criteria for EOSS stage 2 or higher. It provides GPs with a structured framework for further assessments to confirm a timely diagnosis in patients who screen positive. It may also help GPs initiate a discussion about the health benefits of weight loss, resulting in improvements in the quality of care and health outcomes for their patients.

^1^Atlantis E. et al, Clinical obesity 10 (4), e12368

^2^Sharma AM & Kushner RF. Int J Obes (Lond). 2009 Mar;33(3):289-95

^3^Atlantis E. et al, Obesity Reviews 21 (11), e13120

The development of the EOSS-2 Risk Tool was enabled by support from iNova Pharmaceuticals (Australia) Pty Limited, in partnership with the National Association of Clinical Obesity Services (NACOS), and Western Sydney University.

The EOSS-2 Risk Screening Tool

1. Have either of your parents, or any of your brothers or sisters been diagnosed with **diabetes (type 1 or type 2)**?

Yes 25 points

No 0 points

1. Have either of your parents, or any of your brothers or sisters been diagnosed with **high blood pressure (hypertension**)?

Yes 25 points

No 0 points

1. Have either of your parents, or any of your brothers or sisters been diagnosed with **high sugar in blood or urine**?

Yes 25 points

No 0 points

1. Have either of your parents, or any of your brothers or sisters been diagnosed with **high cholesterol levels**?

Yes 25 points

No 0 points

1. How much **bodily pain** have you experienced during the last four weeks?

None 0 points

Very mild to mild 0 points

Moderate 25 points

Severe to very severe 25 points

1. Do you have any form of **disability or limitation or long term condition** that impacts your daily life? If yes, which of these options best describes your status?

Has no core limitation 0 points

Has mild core‎ activity limitation 0 points

Has moderate core activity limitation 25 points

Has severe core activity limitation 25 points

Has profound core activity limitation 25 points

1. What is your **age group**?

18-24 years 0 points

25-34 years 2 points

35-44 years 3 points

45-54 years 5 points

55-64 years 6 points

1. In general, how would you rate your **health and quality of life**?

Excellent 0 points

Very good 3 points

Good 5 points

Fair 8 points

Poor 25 points

1. Have you had any **history of depression or anxiety**?

Yes 10 points

No 0 points

Add up your points

Your risk (chance) of having weight related health problems:

- <7 points: High risk – 42% chance
- 7-24 points: Very high risk – 67% chance
- ≥25 points: Extremely high risk – 89% chance
