# Supplementary material for: Clinical usefulness of brief screening tool for activating weight management discussions in primary cARE (AWARE): A nationwide mixed methods pilot study
Source: PLoS One. 2021 Oct 28;16(10):e0259220. doi: 10.1371/journal.pone.0259220 (PMC8553075; doi:10.1371/journal.pone.0259220)
Supplement: S2 Appendix — (DOCX) [file pone.0259220.s002.docx]

**S2 Appendix. Interview schedules**

**Pilot Study Interview Schedule for GPs**

**Relevance**

- Can you tell me why you were interested in participating in this study?

**Experience/Efficiency**

- Can you tell me about your experiences of using the new EOSS-2 risk screening tool in your practice?
- Can you tell me what aspects of the tool worked well / did not work well?
- Can you tell me about the barriers (if any) to using the EOSS-2 risk screening tool?

**Effectiveness**

- Did you feel comfortable using the EOSS-2 risk screening tool? Why? Why not?
- Can you tell me about your perceptions of the tool in terms of its effectiveness in initiating discussions about weight-related complications?
- Can you tell me about your perceptions of the tool in terms of its effectiveness in initiating weight-management plans?
- After using the screening tool, can you share your perceptions about whether it was effective for any or all of your patients?
- If you perceive it is more effective for certain groups of people, can you elaborate?

**Sustainability**

- Would you consider using the EOSS-2 risk screening tool long-term? Why / Why not?

**Pilot Study Interview Schedule for Patients**

**Relevance**

- Can you tell me why you were interested in participating in this study?

**Experiences of having the screening tool applied/participating in the study**

- Can you tell me about your experiences of participating in the study?
- What aspects were good / not so good?

**Effectiveness**

- Since consenting to participate, have you had a discussion with your GP about weight related issues? If so, how did you feel about the discussion?
- Has your understanding of weight related health problems changed since participating in this study? If so, how?
- How has the discussion with your GP impacted on your confidence in being able to manage your weight?
- Did your GP initiate a weight management plan? If so, how did that make you feel?
- Has the discussion with your GP changed your motivation to improve your health? If so, how?

**Sustainability**

- Has the discussion with your GP changed your motivation to adopt long-term strategies to improve your health? If so, how?
- How do you perceive participation in this study will impact on your health into the future?
