# Supplementary material for: Clinical usefulness of brief screening tool for activating weight management discussions in primary cARE (AWARE): A nationwide mixed methods pilot study
Source: PLoS One. 2021 Oct 28;16(10):e0259220. doi: 10.1371/journal.pone.0259220 (PMC8553075; doi:10.1371/journal.pone.0259220)
Supplement: Attachment — Submitted filename: Response to Reviewers_2.docx [file pone.0259220.s003.docx]

**Response to Reviewers**

**Editor:**

1. We strongly discourage the unnecessary division of related work into separate manuscripts, and we will not consider manuscripts that are divided into parts. Each submission to PLOS ONE must be written as an independent unit and should not rely on any work that has not already been accepted for publication.

If your manuscript cannot be reviewed and published as an independent unit, please remove this submission and the related submission by selecting “Remove Submission” under the Action links in Editorial Manager. Then combine the manuscripts, submit them as one new submission, and indicate in the Previous Interactions field that you have combined your previous submissions.

*The manuscript is an independent unit and does not rely on any work that has not already been accepted for publication. We have removed the reference to work that has not already been accepted for publication, and I confirm that our manuscript can reviewed and published as an independent unit.*

If your manuscript is an independent unit, please remove from your title the reference to the related submission, i.e., (“Part 1,” “Part III,” etc.) Please make sure that the new title will distinguish it from the other submission(s).

*There is no reference in the title to any related submission.*
